# New Books

**Published:** 1877-01-20

**Authors:** 


### BOOKS.

MEDICAL DIAGNOSES—With Special Reference to Practical Medicine. A Guide to the Knowledge and Discrimination of Diseases. By J. M. DA COSTA, M. D., Professor of the Practice of Medicine at the Jefferson Medical College, Philadelphia, etc., etc. Illustrated with Engravings on Wood. Fourth Edition. Revised. Philadelphia. J. B. LIPPINCOTT & Co., 1876.

This valuable work has been before the profession for some years. The present edition is much improved over former ones, by the many changes and additions which have been demanded in order to keep the subject matter abreast with the recent advances in pathology, etc. Diagnosis is the basis upon which all successful practice must rest. The practitioner, well grounded in anatomy, physiology, pathology, and materia-medica, will find Dr. Da Costa's volume one of the best helps in aiding him in attaining success in his practice. In our judgment, no practitioner should be without this helpful volume, and deprives himself of much when he fails to have it on hand for consultation. We advise all to obtain a copy. Like everything else from the publishing house of J. B. Lippincott & Co., the letter-press of this work is all the most fastidious could require. G.

FILTH DISEASES AND THEIR PREVENTION. By JOHN SEMON, M.D., F. R. C. S., Chief Medical Officer of the Privy Council and of the Local Government Board of Great Britain. First American Edition. Printed under the direction of the State Board of Health of Massachusetts. Boston. JAMES CAMPBELL, 1876. Price $1.

There is scarcely a subject about which so little is known as that of Filth Diseases. Everywhere where man finds a "local habitation" filth diseases prévail—whether in the home, city, or rural sites. It matters not how isolated may be his home, these diseases are liable to occur. The importance of their prevention cannot be over-estimated, particularly as the diseases so caused are the most common and fatal to which man is subject. The prevention recommended does not lie in medication—not in preventive medicine—but in respect to a knowledge of correct sanitation, based upon the laws of hygiene. This little volume will be of immense service to the people, if its teachings are properly sustained and enforced by the example and practice of medical men. But the profession, as a mass, are not sufficiently well grounded in the principles of hygiene, and, until they bocome well informed, can never give wholesome advice upon filth diseases and their prevention. Among the valuable helps in obtaining information on so important a subject the little volume above will be useful and helpful. G.

SCRIBNER'S MONTHLY. — Have you ever subscribed for Scribner? If not a treat is in store for you when you do. It will do you good—your wife, if you have one, good—and your family good. Dr. Holland's beautiful story, Nicholas Menturn, is worth the subscription to say nothing of the attractive illustrations with which it is filled, and the quantity of other valuable reading in it. Price $4. G.

ST. NICHOLAS.—Did your child ever see St. Nicholas? If not, you have deprived it of one of the greatest pleasures it is possible for it to have in life. St. Nicholas is the best, purest, the finest illustrated, and most entertaining child's magazine in the world. Let your little ones have it. It costs but $3 a year. G.

